# Introducing two types of psychological resilience with partly unique genetic and environmental sources

**DOI:** 10.1038/s41598-021-87581-5

**Published:** 2021-04-21

**Authors:** Live Skow Hofgaard, Ragnhild Bang Nes, Espen Røysamb

**Affiliations:** 1 Department of Psychology, Promenta Research Centre, University of Oslo, Oslo, Norway; 2 The Norwegian Institute of Public Health, Oslo, Norway

**Keywords:** Psychology, Health care

## Abstract

Psychological resilience is indicated when individuals demonstrate good mental health despite exposure to significant stress or adversity. Good mental health may involve low levels of illbeing and/or high levels of wellbeing. There is still very limited knowledge about the potential differences between these outcomes in relation to stressors. We propose a distinction between type 1 and type 2 resilience, examine their underlying genetic and environmental architecture, and identify modifiable resilience factors. The data come from a population-based twin sample (N = 1987, mean age = 63) in the Norwegian Twin Registry. Type 1 and type 2 resilience are operationalised as the residual of anxiety/depression symptoms and life satisfaction, respectively, after lifetime cumulative adversity has been regressed out. We used biometric modelling and cotwin-control linear mixed models to estimate underlying factors and identify predictors while controlling for genetic confounding. The results support the notion of two separate, but partly overlapping types of resilience. We find heritabilities of 0.30 (type 1) and 0.24 (type 2) and a genetic correlation of 0.43. Potentially causal resilience factors include, but are not limited to, meaning in life, physical activity, positive affect and relationship satisfaction. Whereas some factors are associated with both resilience types, other factors are unique to each type.

## Introduction

Resilience has been defined as positive adaptation despite significant stress or adversity^1^. It is commonly seen as a dynamic *process* unfolding during and after adversity, or the *outcome* of this process^1–5^. When conceptualised as an outcome, resilience can be operationalised as good mental health in relation to stressor load^6–8^.

Resilience studies may usefully inform mental health workers and governments on ways to protect the mental health of individuals in diverse contexts of risks, such as chronic adversity (e.g., poverty), single-incident traumas (e.g., natural disasters, terror attacks) and cumulative adversity. Stress-related mental health problems cause widespread human suffering and are associated with great individual and societal costs^9^. By contrast, mental wellbeing confers considerable advantages to both mental and somatic health, productivity and longevity^10–12^. Thus, knowledge on factors that may contribute to prevent mental health problems and sustain wellbeing despite risks for negative development, can have implications at both an individual and societal level.

When operationalising resilience, researchers have used different conceptualisations of mental health. Some resilience researchers have focused on wellbeing, such as life satisfaction or positive affect^13,14^, others on mental health problems, such as symptoms of PTSD, depression or anxiety^15,16^. If mental wellbeing and illbeing represent opposite poles on the same scale, as suggested by some researchers^17^, these previous studies essentially focus on different aspects of the same dimension. However, wellbeing and illbeing may reflect separate, but related, unipolar dimensions. Indeed, studies suggest that wellbeing (i.e., emotional-, psychological- and social wellbeing, positive affect, life satisfaction) and illbeing (i.e., psychiatric disorders, internalising symptoms, negative affect) have only moderate phenotypic overlap^18,19^ and are affected by partly independent genetic and environmental sources^20–24^. In other words, the presence of wellbeing does not necessarily involve the absence of illbeing, and the genetic and environmental influences that contribute to greater wellbeing are not merely the same as those that contribute to less illbeing. Consequently, resilience studies using different conceptualisations of mental health, could essentially give information about different, though related, outcomes. Failing to address this point, valuable information about potentially different predictors and mechanisms for the two types of adaptation may be precluded or entirely lost.

To take a closer look at this potential issue, we introduce a distinction between type 1 and type 2 resilience, focusing on illbeing and wellbeing, respectively, in the context of stressors. Type 1 resilience involves sustained, regained or decreased levels of illbeing (less illbeing than expected), while type 2 resilience involves sustained, regained or increased levels of wellbeing (more wellbeing than expected), despite significant stress or adversity. This division is relevant when conceptualising positive adaptation as good mental health (as opposed to e.g. the accomplishment of salient developmental tasks). It adds to already established resilience terms such as emergent and minimal-impact resilience^25^, that have to do with the stressor in focus (chronic adversity vs single-incidence trauma) rather than the chosen outcome-variable in the operationalisation of resilience.

Stressor load can be measured by the sum of adverse life events experienced. Previous studies have demonstrated a linear and negative effect of cumulative adverse life events (e.g., maltreatment, conflicts in close relationship, serious accidents or illness) on wellbeing^26^ and a similar positive effect on mental illbeing^27^. However, some individuals show less illbeing and/or more wellbeing than expected given their cumulative adversity. On this background, Amstadter et al. introduced an approach to quantify resilience based on a linear regression of the relationship between cumulative adversity and mental health^15^. A high regression residual would indicate better mental health than predicted (i.e., high resilience). Amstadter et al.^15^ used a past-year framework for self-reported adversities, but the same method could potentially be used when applying a lifetime perspective. However, it is not clear whether the relationship between adversities and mental health remains linear or becomes curvilinear^28^ when including adversities throughout life.

In addition to its effect on mental health, lifetime cumulative adversity seems to have negative effects on age-related changes in cognitive and physical health, that accelerate if the individual’s mental health is poor^29,30^. In other words, facilitating resilience to lifetime cumulative adversity (i.e., good mental health despite this risk), may further decelerate age-related cognitive and physical declines. Hence, resilience to lifetime cumulative adversity seems especially relevant to study in the population of adults beyond midlife who have lived a long life and are entering a developmental phase where age-related declines become more prominent. The study of resilience in this age group may help to discover potentially age-specific variations which, in turn, can usefully inform interventions.

In order to facilitate resilience to lifetime cumulative adversity, we need to know the underlying causes. For one, individuals may differ in their genetic potential for a strong “psychological immune system”. Indeed, research indicate that genetic factors affect our stress-response system and how sensitive we are to environmental influences^5,31,32^. Interestingly, the genetic influences underlying environmental sensitivity to negative experiences seem to be somewhat different from those affecting the sensitivity to positive experiences^31^. In other words, some genetic factors may contribute to resilience by protecting against the influence of negative experiences, and others may compensate for the effect of negative events by providing specific sensitivity to positive experiences. Recently, genome-wide association studies (GWASs) have contributed to further development in the field^32,33^, and one study has identified three specific loci related to resilience^34^.

Although few behavioural genetic studies have been conducted on resilience among adults, twin studies indicate that genetic variation explains between 25 and 52% of the variation in resilience^13,15,35,36^. Wolf et al. (2018) estimated a heritability of 0.25 for questionnaire-based resilience in a sample of male twin pairs from the Vietnam Era Twin Registry^35^. Somewhat higher heritability estimates of 0.52 for men and 0.38 for women were reported by Boardman et al. when conceptualising resilience as the residual of *positive affect* after controlling for social and interpersonal stressors^13^. Furthermore, a longitudinal twin study by Amstadter et al.^15^ looked at the heritability of resilience operationalised as the difference between actual and predicted *internalising symptoms* in relation to cumulative adversity. They reported a general heritability estimate of 0.31 for each wave over a 5-year period. Of note, the heritability was estimated to 0.50 when specifying a latent variable and incorporating error of measurement into the model^15^. In another study based on bivariate genetic modelling, using the same operationalisation of resilience, Amstadter et al. reported a heritability estimate between 0.33 and 0.34^36^. The studies by Boardman et al.^13^ and Amstadter et al.^15,36^ used a past-year framework for self-reported adversities. To our knowledge, there have been no studies looking at the heritability of resilience to lifetime cumulative adversity among adults.

The twin studies above indicate a role for genetic factors, but they also highlight that much of the individual variation in resilience seems to be due to environmental influences. These may be direct influences, as the environment in which we live may facilitate or obstruct resilience^37^, but may also be indirect, operating through our individual characteristics. Indeed, both genetic and environmental influences affect individual differences in most psychological traits^38^, some of which may contribute to resilience.

Several studies have indicated individual and social resources associated with resilience among adults. Socioeconomic factors, such as educational background, have been related to resilient trajectories when facing natural disasters^39^ and the onset of disability in later life^40^. Also, physical health seems to play a role. Physical fitness has been shown to moderate the relationship between negative life events and depression symptoms^41^, and one experimental study on healthy adults demonstrated how regular physical activity protected against declines in positive affect in response to a stress-test^42^. Another study suggests that how we evaluate our own physical health is even more important than our actual physical activity when it comes to coping with everyday stressors^43^.

Moreover, the availability of social support has been related to positive mental health outcomes in the context of stress and adversity^44^. For instance, social support measured before the Great East Japan earthquake and tsunami reduced the chance for post-disaster depression among adults aged 65 and above^45^. In contrast, the experience of loneliness has been associated with negative stress-responses^44^. Furthermore, an experimental study demonstrated that holding the husband’s hand, compared to a stranger’s hand or no hand at all, reduced women’s threat-response when facing electric shock, with the effect varying as a function of relationship satisfaction^46^. Relatedly, adult attachment style has been linked to resilience. For instance, secure attachment (among other variables) predicted low psychological distress despite high prevalence of lifetime cumulative adversity among war veterans^47^. It also predicted lower stress response (measured as affected HPA reactivity and alteration of gene expression) to prolonged isolation and confinement in a longitudinal study^48^. Additionally, high level of social trust is associated with reduced negative effects of adversities (discrimination, ill-health, unemployment) on life satisfaction in adult populations across several European countries^49^.

Other resources related to resilience are self-efficacy, optimism, positive affect and a sense of purpose or meaning in life. Self-efficacy (i.e., the perceived capacity to handle difficulties) may protect against negative psychological effects of diverse challenges such as perceived stress^50^, being diagnosed with cancer^51^, and living with spinal cord injury^52^. One potential explanation is that individuals high in self-efficacy tend to apprise stressful situations as challenging (giving rise to effort) rather than threatening or harmful, even in conditions of continuous failure experiences^53^. Optimism (i.e., positive expectations and positive explanatory style) seems to moderate the effect of stressful life events on depression and anxiety symptoms^54^. It has also been linked to lower levels of posttraumatic stress symptoms among survivors of a deadly earthquake^55^, and to positive psychological health (i.e., life satisfaction, positive affect) among repatriated prisoners of war followed over a 40-year period^56^. Furthermore, higher average levels of positive affect appears to reduce the impact of pain and perceived interpersonal stress on negative affect^57^. Similarly, the ability to generate boosts of positive affect from daily events (high reward experience) seems to moderate the impact of childhood adversity and recent stressful life events on later symptoms of depression and anxiety^58^. Finally, having a sense of meaning in life seems to aid resilience, as demonstrated in studies on war veterans looking at resilience to the effect of lifetime cumulative adversity on symptoms of posttraumatic stress, depression and anxiety^47,59^.

This list is not exclusive but represents selected key factors previously associated with resilience and stress-responses across diverse situations. Examining all these factors together may help to compare effects and identify potentially unique contributions to type 1 and/or type 2 resilience in the context of lifetime cumulative adversity.

Noteworthy, the abovementioned studies are mostly observational. Consequently, unobserved factors, such as shared environmental or genetic effects, may affect the associations. If, for example, a predictor and resilience are affected by the same genetics, they will associate in analyses, even if there is no effect of the predictor on resilience. Environmental efforts (i.e., interventions, social policies) to influence such predictors are likely to be futile with regard to resilience and may result in more economic pain than mental gain. Indeed, modest overlap in genetic influences between resilience and related factors (e.g., optimism) has been demonstrated^36^, underlining the importance of controlling for genetic confounding when examining the effect of such factors on resilience. One way to do this is through the use of a cotwin-control design, comparing monozygotic (MZ) twins reared together, who share 100% of their genetic material. This allows for estimating environmental effects while controlling for shared genetic and familial factors, and thus helps to identify potentially modifiable resilience factors. Few resilience studies to date have utilised such designs, with one recent exception looking at the effect of conscientiousness on the relation between low socioeconomic status and later life outcomes^60^.

To sum up, although resilience research has flourished the last decades, there is still much to be discovered and a need for further clarity, both with regards to the conceptualization of resilience and its etiological underpinnings. The current study attempts to address important perspectives highlighted in this introduction that until now have remained inadequately studied in the research literature.

Comparing two potentially different types of resilience, using a sample of almost 2000 twins beyond midlife (56–71 years old), we aim to (1) investigate overlap and specificity of type 1 and type 2 resilience; (2) disentangle the genetic and environmental influences on the resilience types and their association; and (3) identify unique and common predictors of type 1 and type 2 resilience when controlling for confounding genetics and shared environment.

## Results

### Descriptives

Descriptive statistics can be seen in Table 1. For correlations between all variables, see Supplementary Table S1.

**Table 1:** Descriptive statistics.

	Number of valid cases	Minimum	Maximum	Mean	Standard deviation
Educational background	1980	1.00	6.00	3.26	1.27
Self-rated health	1977	1.00	5.00	4.08	0.80
Physical activity	1951	1.00	5.00	3.85	1.26
Relationship satisfaction	1620	1.00	6.00	4.92	0.90
Social support	1971	1.00	3.00	2.38	0.48
Trust	1965	0.67	10.00	7.33	1.66
Anxious attachment	1776	1.00	6.67	2.67	1.12
Avoidant attachment	1769	1.00	7.00	2.31	1.09
Loneliness	1927	1.00	5.00	1.73	0.73
Positive affect	1971	1.00	5.00	3.58	0.67
Meaning in life	1956	0.00	10.00	8.08	1.64
Optimism	1970	1.00	5.00	3.94	0.69
Self-efficacy	1954	1.00	4.00	2.93	0.65
Type 1 resilience	1910	− 3.58	2.40	0.00	1.00
Type 2 resilience	1962	− 2.98	3.35	0.00	1.00
Lifetime stressors/adversities	1987	0.00	12.00	2.19	1.95
Satisfaction With Life Scale (SWLS)	1962	1.00	7.00	5.39	1.16
Symptom checklist (SCL-8)	1910	1.00	4.00	1.23	0.39
Birth year (age)	1987	1945	1960	1953	4.50

### Type 1 and type 2 resilience

#### Operationalization

Our outcome-approach to operationalising resilience involves two assumptions: (1) individuals vary in their sum of reported stressful life events, and (2) lifetime cumulative adversity is associated with the level of internalising symptoms and life satisfaction. Simple linear analyses confirmed that there are individual differences in the sum of reported stressful life events, and that anxiety/depression symptoms increases (*r* = 0.33, p < 0.001) and life satisfaction decreases (*r* = − 0.31, p < 0.001) with the number of total stressful life events reported (see Supplementary Figs. S1 and S2). We tested whether a curvilinear relationship would prove a better fit, but the results were essentially the same (see Supplementary Figs. S3 and S4). The standardised residuals from the linear regression analyses were saved and represent resilience to the effects of lifetime cumulative adversity (independent variable) on illbeing (type 1 resilience) and wellbeing (type 2 resilience), respectively. Type 1 resilience was reversed.

#### Overlap and uniqueness

The phenotypic correlation analysis indicated some overlap, but also distinct variation for type 1 and type 2 resilience (*r* = 0.45, p < 0.001). This observation made it relevant to look closer at the differences and similarities in sources and predictors for the two resilience types.

### The relative contribution of genetic and environmental influences

#### Twin correlations

Table 2 displays the phenotypic (within-twin) correlations, the cross-twin correlations and the cross-twin cross-trait correlations by zygosity. The considerably stronger pair resemblance for MZ than DZ twins, with rDZ about half the size of rMZ for both resilience types, is suggestive of additive genetic factors.

**Table 2:** Twin correlations for type 1 and type 2 resilience: within-twin correlations, cross-twin correlations and cross-twin cross-trait correlations by zygosity.

	n (twins)	Within-twin	Cross-twin: type 1 resilience	Cross-twin: type 2 resilience	Cross-twin cross-trait
MZ	857	0.44	0.33	0.22	0.12
DZ	1041	0.45	0.14	0.11	0.07
MZ_m_	355	0.46	0.33	0.24	0.14
MZ_f_	502	0.42	0.32	0.21	0.11
DZ_m_	439	0.48	− 0.04	0.10	− 0.01
DZ_f_	602	0.44	0.21	0.12	0.10

#### Biometric modelling

Bivariate biometric modelling was conducted to estimate genetic and environmental contributions to variance and covariance for type 1 and type 2 resilience. A Full (ACE) model (Model 1) was tested against five nested models with different numbers of parameters (ACE; AE) and varied inclusion of sex differences (see the “Methods” section for further details). Fit statistics for the nested models are shown in Table 3.

**Table 3:** Model-fitting results from the bivariate analyses.

Model no	Model	Minus2ll	df	AIC	Δχ^2^	$\Delta_{df}$
1	Full ACE	20,897.747	7722	5453.7469	NA	NA
2	AE_csl	20,898.563	7728	5442.5634	0.8165	6
3	ACE_scalar	20,901.813	7730	5441.8132	4.0663	8
4	**AE_scalar**	**20,903.447**	**7736**	**5431.4466**	**5.6997**	**14**
5	ACE_nsl	20,960.283	7731	5498.2826	62.5357	9
6	AE_nsl	20,960.283	7734	5492.2826	62.5357	12

Best fitting model in bold type.

*Csl* common sex limitations (allow for quantitative sex differences), *scalar* scalar sex limitations, *nsl* no sex limitations. Total N = 1987 twins.

**Figure 1:**
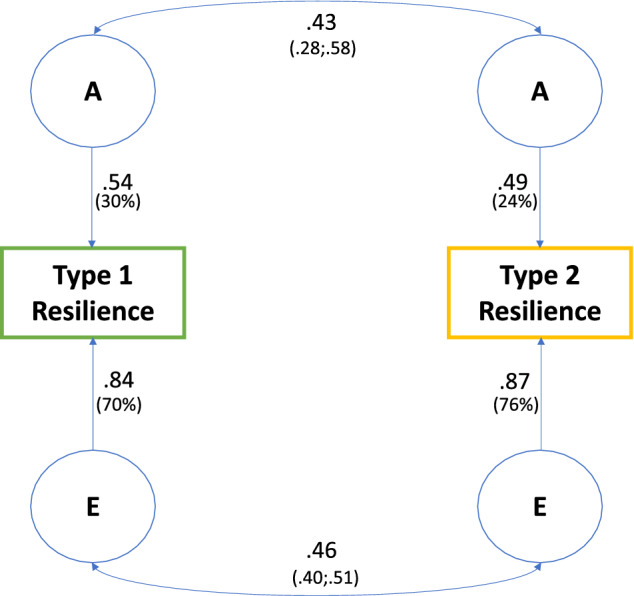
Results from the best fitting bivariate model. “A” indicates the latent additive genetic factors and “E” represents individual-specific environmental factors. The magnitude of each path is shown in the figure, including the percent of the variance in the observed variable accounted for by the latent factors.

The best fit was given by Model 4, the AE model with no gender differences in heritability (scalar sex limitation). This model had the lowest AIC value and the minus2LL was not significantly poorer than for Model 1. Based on this best-fitting model, the standardised parameters were calculated, as well as the genetic and environmental correlations. The resulting heritability estimates (with 95% CIs) were 0.30 (0.24; 0.37) for type 1 resilience and 0.24 (0.17; 0.30) for type 2 resilience, with the remaining 0.76 and 0.70, respectively, attributed to individual-specific environmental variation. The correlation between the additive genetic factors influencing type 1 and type 2 resilience (r_g_) was estimated to 0.43 (0.28; 0.58). The corresponding correlation for the individual-specific environmental influences (r_e_) was estimated to 0.46 (0.40; 0.51). The proportions of the phenotypic covariance attributed to genetic and environmental similarities were estimated to 25.5% and 74.5%, respectively. Figure 1 portrays the best-fitting model with path coefficients from the additive genetic factors and the individual-specific environmental factors, as well as the estimated correlations between additive genetic factors and individual-specific environment.

### Predictors

Predictors of the two resilience types were investigated in regression analyses using a co-twin control method to account for shared genetics (no effect of shared environment was found). Five different models were tested. Model 1 involves univariate regression analyses for each variable separately with control for age and biological sex. Model 2–4 include a co-twin control, where Model 3 has an additional control for the other resilience type, to test potentially unique contributions, and Model 4 is a multivariate Model. The results for type 1 and type 2 resilience can be seen in Tables 4 and 5.

**Table 4:** Regression analyses for type 1 resilience.

Variables	Model 1		Model 2		Model 3		Model 4	
	β	p	β	p	β	p	β	p
Educational background	**0.16**	**0.00**	0.02	0.79	− 0.01	0.86		
Self-rated health	**0.33**	**0.00**	**0.26**	**0.00**	**0.12**	**0.04**	**0.16**	**< 0.05**
Physical activity	**0.11**	**0.00**	**0.19**	**0.00**	**0.16**	**0.00**	**0.17**	**< 0.01**
Relationship satisfaction	**0.27**	**0.00**	**0.22**	**0.00**	0.03	0.71	0.00	> 0.05
Social support	**0.11**	**0.00**	**0.15**	**0.02**	0.10	0.07	0.03	> 0.05
Trust	**0.21**	**0.00**	**0.18**	**0.00**	**0.14**	**0.01**	0.04	> 0.05
Anxious attachment	**− 0.29**	**0.00**	− 0.12	0.06	− 0.01	0.85		
Avoidant attachment	**− 0.28**	**0.00**	**− 0.21**	**0.00**	− 0.08	0.19	− 0.08	> 0.05
Loneliness	**− 0.31**	**0.00**	**− 0.29**	**0.00**	**− 0.16**	**0.00**	− 0.10	> 0.05
Positive affect	**0.38**	**0.00**	**0.35**	**0.00**	**0.16**	**0.00**	**0.14**	**< 0.05**
Meaning in life	**0.44**	**0.00**	**0.39**	**0.00**	**0.20**	**0.00**	0.10	> 0.05
Optimism	**0.37**	**0.00**	**0.28**	**0.00**	**0.15**	**0.00**	0.04	> 0.05
Self-efficacy	**0.30**	**0.00**	**0.26**	**0.00**	**0.12**	**0.02**	0.08	> 0.05

Significant effects (p < 0.05) in bold type.

N varies between 415 and 888. See Method section and Table 1 for more information.

Model 1: Linear model, standardized, with control for sex and age, for each variable separately.

Model 2: Linear mixed model, standardized, with control for sex, age and genetic confounding, for each variable separately.

Model 3: Linear mixed model, standardized, with control for sex, age, genetic confounding and the other resilience measure, for each variable separately.

Model 4: Multivariate linear mixed model, standardized, with control for sex, age and genetic confounding, with all significant variables from Model 2 included.

**Table 5:** Regression analyses for type 2 resilience.

Variables	Model 1		Model 2		Model 3			Model 4		
	β	p	β	p	β		p	β		p
Educational background	**0.12**	**0.00**	0.08	0.40	0.05		0.50			
Self-rated health	**0.41**	**0.00**	**0.37**	**0.00**	**0.27**		**0.00**	**0.20**		**< 0.01**
Physical activity	**0.09**	**0.01**	**0.13**	**0.03**	0.01		0.90	0.07		> 0.05
Relationship satisfaction	**0.44**	**0.00**	**0.49**	**0.00**	**0.46**		**0.00**	**0.23**		**0.00**
Social support	**0.13**	0**.00**	0.08	0.22	0.03		0.58			
Trust	**0.21**	**0.00**	0.11	0.06	0.04		0.48			
Anxious attachment	− **0.27**	**0.00**	− **0.26**	**0.00**	− **0.21**		**0.00**	0.06		> 0.05
Avoidant attachment	− **0.35**	**0.00**	− **0.33**	**0.00**	− **0.24**		**0.00**	− 0.02		> 0.05
Loneliness	− **0.31**	**0.00**	− **0.33**	**0.00**	− **0.23**		**0.00**	− 0.09		> 0.05
Positive affect	**0.56**	**0.00**	**0.54**	**0.00**	**0.43**		**0.00**	**0.26**		**0.00**
Meaning in life	**0.62**	**0.00**	**0.59**	**0.00**	**0.49**		**0.00**	**0.30**		**0.00**
Optimism	**0.35**	**0.00**	**0.33**	**0.00**	**0.23**		**0.00**	0.02		> 0.05
Self-efficacy	**0.36**	**0.00**	**0.35**	**0.00**	**0.25**		**0.00**	**0.13**		**< 0.05**

Significant effects (p < 0.05) in bold type.

N varies between 415 and 888. See Method section and Table 1 for more information.

Model 1: Linear model, standardized, with control for sex and age, for each variable separately.

Model 2: Linear mixed model, standardized, with control for sex, age and genetic confounding, for each variable separately.

Model 3: Linear mixed model, standardized, with control for sex, age, genetic confounding and the other resilience measure, for each variable separately.

Model 4: Multivariate linear mixed model, standardized, with control for sex, age and genetic confounding, with all significant variables from Model 2 included.

#### Type 1 resilience

For type 1 resilience, Model 1 indicated significant contributions at the p < 0.01 level for all variables. When including the co-twin control (Model 2), 11 variables remained significant (p < 0.05): self-rated health, physical activity, relationship satisfaction, social support, trust, avoidant attachment, loneliness, positive affect, meaning in life, optimism, and self-efficacy. When further controlling for type 2 resilience (Model 3), relationship satisfaction, social support and avoidant attachment lost their significance. The multivariate regression analysis in Model 4 indicated only three significant variables (p < 0.05): self-rated health, physical activity and positive affect. For Model 4, the ${R}^{2}$ was estimated to 29%.

#### Type 2 resilience

In Model 1 all variables significantly predicted type 2 resilience (p < 0.01). However, in the co-twin control analyses (Model 2), only the following factors remained significant (p < 0.05): self-rated health, physical activity, relationship satisfaction, anxious attachment, avoidant attachment, loneliness, positive affect, meaning in life, optimism and self-efficacy. With the exception of physical activity, these variables were also significant (p < 0.01) in Model 3 with the additional control for type 1 resilience. When looking at the multivariate model (Model 4), only self-rated health, relationship satisfaction, positive affect, meaning in life and self-efficacy were significant (p < 0.05). For Model 4, the ${R}^{2}$ was estimated to 50%.

**Figure 2:**
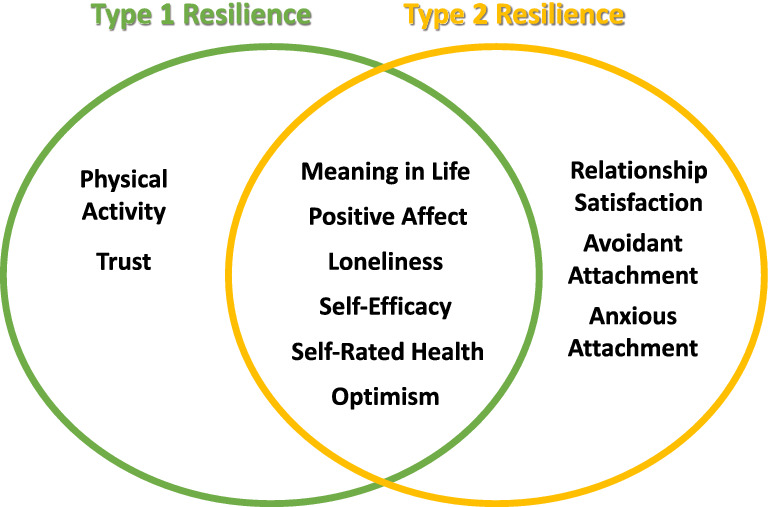
An illustration of the cotwin-control results with significant predictors (p < 0.05), based on Model 3. Hence, the illustration indicates unique and overlapping predictors for both types of resilience, unconfounded by shared environment and genetics.

Figure 2 summarises the results from Model 3 for both resilience measures.

## Discussion

We introduced a novel conceptualisation of type 1 and type 2 resilience to explore potential differences between positive mental health outcomes (less illbeing than expected vs. more wellbeing than expected) in the context of stressors and adversities. The resilience types were operationalised as the residual of anxiety/depression symptoms and life satisfaction, respectively, after lifetime cumulative adversity had been regressed out. Our results support three conclusions: (1) it is meaningful to distinguish between type 1 and type 2 resilience; (2) there is a moderate genetic contribution to the two resilience types and their association; and (3) there are both shared and unique predictors of type 1 and type 2 resilience. The findings underlying these conclusions will now be discussed.

First, the phenotypic correlation of 0.45 indicates the presence of two different, but partly overlapping, types of resilience. Hence, at a given point in time one may display both outcomes of resilience (i.e., more wellbeing and less illbeing than predicted), just one of them, or neither. Furthermore, the estimated genetic correlation between type 1 and type 2 resilience was 0.43, and the individual-specific environmental correlation was 0.46. Accordingly, some genetic and environmental factors may aid both forms of resilience, while other factors may benefit either type 1 or type 2 resilience. The moderate genetic correlation is in line with previous reports on the relation between illbeing and wellbeing^20–24^. However, the current estimates represent residuals after lifetime adversities have been regressed out. Thus, they specify genetic effects after control for the genetics that may influence both the exposure to adverse life events (selection into events) and the mental health outcomes.

Moreover, the heritability coefficients were estimated to 0.30 and 0.24 for type 1 and type 2 resilience, respectively. The remaining variation (0.70 for type 1 and 0.76 for type 2) was explained by individual-specific environmental sources and measurement errors. There was no effect of biological sex on the heritability estimates in our data, which is in line with Amstadter et al., but contrasts the findings of Boardman et al. who suggest that heritability plays a larger role in resilience for males than for females. Also, in accordance with previous twin research on resilience among adults^13,15,36^, we found no significant effect of shared environmental influences. This suggests that resilience among adults relies on genes and individual-specific environmental experiences rather than previous family environment shared by siblings.

The moderate heritability estimates are similar to those of Amstadter et al.^15,36^, focusing on the equivalent of type 1 resilience (h^2^ = 0.31–0.34, without the control for measurement errors and occasion-specific effects), and Boardman et al.^13^, focusing on the equivalent of type 2 resilience (h^2^ = 0.38 for women and 0.52 for men). Thus, the heritability of resilience to lifetime cumulative adversity may not be much different from that of cumulative adversity in the past year alone. Still, the estimates are slightly lower in our data, especially for type 2 resilience. This may indicate that genes have somewhat less effect in protecting our wellbeing when looking at stressors in a lifetime perspective. Alternatively, the variation may be due to different outcome variables to indicate wellbeing (life satisfaction in our study and positive affect in Boardman et al.’s study), or the diverse age groups in focus (mean age of 63 in our study compared to 44 in Boardman et al.’s study). The latter would suggest a smaller relative role of genetics in type 2 resilience among older compared to younger adults, possibly due to broader life experiences (i.e., greater environmental variation). Indeed, heritability has been shown to decrease from younger to older adulthood when looking at, for instance, personality traits^61^.

Overall, our results indicate a major role of environmental effects: environmental variation explains most of the total variation in both type 1 and type 2 resilience, and the environmental contribution to the phenotypic overlap between the resilience types is much greater than that of genetic factors (explains 74,5% versus 25.5% of the phenotypic overlap, *r* = 0.45). This fits the notion of the social ecological theory by Ungar, which suggests an important role for environmental influences in resilience^37^. It also emphasises the potential for environmental efforts to facilitate both resilience types.

To identify factors with potentially modifiable environmental effects on resilience, we studied the influence of individual and social resources using a co-twin control design. When controlling for genetic confounding, age and biological sex, as well as the other resilience measure (Model 3), the factors most relevant to type 1 resilience were (decreasingly): meaning in life, physical activity, loneliness, positive affect, optimism, trust, self-efficacy and self-rated health. Using the same model, the factors most relevant to type 2 resilience were (decreasingly): meaning in life, relationship satisfaction, positive affect, self-rated health, self-efficacy, avoidant attachment, optimism, loneliness and anxious attachment.

Our findings support the relevance of these factors for resilience among adults, as suggested by previous reports^41–43,46–52,54–59^, but also add new perspectives. First, they demonstrate how the associations between these factors and resilience remain significant after control for genetic confounding. Second, our findings indicate that some of these factors are common to both resilience types (i.e., finding meaning in daily activities, a perception of good health, experiencing positive affect, being optimistic, not feeling lonely, and believing in one’s capacity to handle difficult demands), while others seem to represent unique effects on either type 1 resilience (i.e., being physically active and trusting others) or type 2 (i.e., feeling satisfied and secure in close relationships)*.*

Noteworthy, relationship satisfaction and avoidant attachment was significantly associated with type 1 resilience after the cotwin-control (Model 2), but not in Model 3, with additional control for type 2 resilience. The same trend was detected for physical activity on type 2 resilience in Model 2 versus Model 3. This could reflect a common resilience factor underlying both resilience types (i.e., relationship satisfaction has an effect on this common factor and an independent effect on type 2 resilience), or it could indicate that the effects of these variables are better explained by the other resilience measure (i.e., the effect of relationship satisfaction on type 1 resilience works through the effect it has on type 2 resilience).

In the multivariate analysis with all significant variables from Model 2 and control for genetic confounding, age and biological sex (Model 4), the remaining significant variables for type 1 resilience were physical activity, self-rated health and positive affect. The corresponding variables for type 2 resilience were meaning in life, positive affect, relationship satisfaction, self-rated health and self-efficacy. This indicates a special relevance of these variables.

Educational background was significantly related to both resilience measures in the univariate regression analyses without control for genetic confounding (Model 1), but was not significant in the co-twin control analyses (Model 2). Hence, the effect of educational background on resilience seems to be confounded by shared genetics. Social support was not significant for type 2 resilience after the co-twin control, but remained significant for type 1 resilience. However, this effect evaporated in Model 3 with control for type 2 resilience. This may indicate that social support shares genetic factors with type 2 resilience specifically.

There are, however, some potential limitations to consider when looking at our results. First, resilience is considered context dependent and the results may be limited to cultural contexts similar to the one in Norway. Second, the outcome-operationalisation of resilience based on lifetime cumulative adversity may have some caveats. The list of adverse life events used to operationalise resilience is not exhaustive and participants may have experienced more adversities than what is reported in our study. Also, we did not control for less adverse daily stressors that may impact our mental health, nor the time that had passed since the events. Moreover, the sum of stressors is not necessarily equal to differences in the burden of stressors. Certain types of stressors may affect mental health more than others (e.g., self-oriented versus other-oriented events)^62^, and the same stressful event item (e.g., serious illness or injury) may refer to very different events for person A (e.g., a sprained arm) than person B (e.g., a severe heart attack)^63^. Besides, possible effects of “toughening”^28^ versus “stress sensitizing”^64^, where previous adversity is associated with decreased or increased sensitivity to later adversity, respectively, could affect the relationship between lifetime adversities and mental health outcomes. Nevertheless, trends in our data indicated that the sum of lifetime adversities affects both SWLS and SCL scores in a linear manner.

Finally, the cross-sectional nature of our study does not allow for conclusions regarding causation. Although the cotwin-control design helps to rule out hypotheses of confounding by unobserved variables such as genes, the direction of effect cannot be fully determined. Longitudinal designs (e.g., growth mixture modelling) are needed to further test the potentially causal effects of the identified factors on resilience. Such designs could also help in determining how and when the different factors affect resilience (i.e., whether they prevent or weaken the negative effect of adversities on mental health, bolster the recovery from adversity-induced mental health disturbances, or improve mental health in general^5^).

Despite some limitations, our results bear theoretical and practical implications. The moderate phenotypic correlation, the partly independent genetic and environmental influences, and the varied roles of both common and unique predictors, underline the importance of distinguishing between type 1 and type 2 resilience. This conceptual differentiation is worth keeping in mind in future research and meta-analyses. It highlights the potential for future studies to look at overlap and uniqueness of the processes that lead to less illbeing and more wellbeing than expected given the stressor load. It may also be relevant to the development and evaluation of health interventions: if only one form of adaptation is assessed, the status on the other type of resilience will be overlooked. Besides, the differentiation may help to recognise different needs in therapy and appreciate signs of not just one, but two different forms of positive adaptation.

Moreover, the results indicate a moderate genetic contribution to the individual variations in type 1 and type 2 resilience, and to the association between them. This underlines the importance of controlling for confounding genetics when looking at possible predictors, and emphasises the relevance for current and future GWA studies to regard both type 1 and type 2 resilience in the search for specific genes.

At the same time, the results indicate a considerable contribution of individual-specific environmental factors. This underscores the potential of interventions and social policy to facilitate resilience: although some individuals may be born more sensitive or robust to stressors than others, most people can acquire skills to boost their “psychological immune system”, and the circumstances can be better shaped to facilitate resilience. For instance, as seen in this study, stimulating a strong sense of meaning in life, encouraging physical activity and focusing on quality relationships, may protect against negative effects of lifetime cumulative adversity on mental health in adults beyond midlife.

## Methods

### Participants

The study involves a population-based twin sample from the Norwegian Twin Registry (NTR). The NTR consists of several cohorts of twins and includes data on zygosity and information on various health-related variables^65^. The current sample includes monozygotic and dizygotic same-sexed twins born between 1945 and 1960. The data were collected in 2016 from 1987 twins (target sample: n = 3090, response rate: 64%), including 571 single and 1416 pair responders, altogether comprising 375 monozygotic males (MzM), 457 dizygotic males (DzM), 528 monozygotic females (MzF) and 627 dizygotic females (DzF). Zygosity was determined by a questionnaire shown to correctly classify 97–98% using genetic marker analysis and DNA-based verification for a sub-sample of twins^66^. The mean age was 63.1 years (SD = 4.5).

The study has been approved by the Regional Committee for Medical and Health Research Ethics of South-East Norway and the Ethical Committee at the Department of Psychology, University of Oslo, Norway. Informed consent was obtained from all participants and all methods were performed in accordance with relevant guidelines and regulations.

### Measures

#### Lifetime cumulative adversity

Lifetime cumulative adversity was calculated based on a standard list of eleven adverse life events adapted from the Norwegian Mother and Child Cohort Study (MoBa)^67^. The list includes the following stressors: problems at work; financial problems; relationship dissolution; problems or conflicts in close relationships (partner and/or family/friends/neighbour); illness or injury (regarding self and/or significant others); exposure to physical violence; serious accident, fire or robbery; maltreatment or abuse; and other traumatic events (open). The response options were “yes”/”no”, within the categories of “the last year” and “previously”, allowing a maximum of 22 stressors in total.

#### Life satisfaction (wellbeing)

The Satisfaction with Life Scale (SWLS)^68^ was used to measure overall life satisfaction. The SWLS is a commonly used measure of life satisfaction worldwide and has been shown to have excellent psychometric properties including validity, internal consistency and test–retest reliability^69^. The scale consists of five items meant to measure global cognitive judgements of life satisfaction, such as “I am satisfied with my life”. Items were rated on a scale from ‘strongly disagree’ (1) to ‘strongly agree’ (7). Cronbach’s α was 0.91.

#### Internalising symptoms (illbeing)

Internalising symptoms of anxiety and depression were measured using an eight-item short version of the Symptom Checklist Anxiety (A) and Depression (D) subscales, originally selected and evaluated for use in the MoBa study^70^. The items include descriptions such as “Feeling fearful” (A) and “Feeling helpless about the future” (D), scored on a 4-point scale from “Not at all” to “very much”. Cronbach’s α was 0.90.

#### Operationalisation of type 1 and type 2 resilience

Inspired by Amstadter et al.^15^, based on the idea that mental health is affected by cumulative adversity in a linear manner, type 1 and type 2 resilience were operationalised as the regression residual of internalising symptoms and life satisfaction, respectively, after the effect of lifetime cumulative adversity had been regressed out. The SCL- and SWLS scores were log-transformed to reduce skewness, and the SCL-score was reversed, before constructing the resilience measures. Hence, for both resilience types, the more positive the residual—the higher the level of resilience.

#### Operationalisation of predictors

Educational background was measured by the highest education level achieved, with the following response options: primary school (1), high school (2), craft/journeyman's certificate (3), bachelor’s degree (4), master’s degree (5), and Ph.D. (6) (standard question, used by e.g. the NTR).

Self-rated health was measured by asking the respondents to rate their general health on a scale from “very good” (1) to “very bad” (5). The scale was reversed in the analyses.

Physical activity was measured by asking the participants to indicate their regularity of physical exercise on a scale from “never” (1) to “3 or more times a week” (5) (standard questions, used by e.g., the NTR and the Norwegian Institute of Public Health, NIPH).

Relationship satisfaction was measured using a short version of the Relationship Satisfaction Scale (RS5)^71^. The RS5 is a 5-item scale with statements such as “I am satisfied with my relationship with my partner”, scored from “strongly agree” (1) to “strongly disagree” (6). Cronbach’s α was 0.87.

Social support was measured using two standard questions adapted from the MoBa study (also used by NIPH and the NTR): “Do you have anyone other than your husband/partner you can ask for advice in a difficult situation?” and “How often do you meet or talk on the telephone with your family (other than your husband/partner and children) or close friends?”. The response options were “No”; “Yes, 1 or 2 people”; and “Yes, more than 2 people”, for the first question, and “Once a month or less”; “2–8 times a month”; and “More than twice a week”, for the second. As social support is understood as a formative index the Cronbach’s α is irrelevant^72^.

Loneliness was measured using three items from the Roberts Version of the UCLA Loneliness Scale (RULS-8)^73^. Statements such as “I feel isolated from other people” were scored on a 5-point scale from “never” to “always”. Cronbach’s α was 0.87.

Trust was rated based on three questions previously used in the European Social Survey (ESS). Items such as “Would you generally say that most people are trusting, or that one cannot be too careful when dealing with others?” were scored on a scale from 0 (little trust in others) to 10 (high degree of trust in others). Cronbach’s α was 0.82.

Adult attachment style was measured by the Experiences in Close Relationship Scale (ECR-N12)^74^. This 12-item scale has two subscales, *Anxious Attachment* and *Avoidant Attachment*, each consisting of six questions regarding one’s emotional insecurity in close relationships (e.g., “I am nervous when partners get too close to me” or “I need a lot of reassurance that I am loved by my partner”). All questions were answered on a 7-point scale from “strongly disagree” to “strongly agree”. Cronbach’s α was 0.77 for Anxious attachment and 0.84 for Avoidant attachment.

Positive affect was measured using selected items from the Differential Emotions Scale (DES)^75^. Three questions about the frequency of happiness and enjoyment in everyday life (e.g., “Feel joyful, like everything is going your way, everything is rosy”) were answered on a 5-point scale from “rarely or never” to “very often”. Cronbach’s α was 0.83.

Meaning in life was measured with one single question: “In general, would you say that what you do in life is meaningful?”, rated from “not meaningful” (0) to “very meaningful” (10) (used in e.g., the European Health Interview Survey, EHIS, and the European Union Statistics on Income and Living Conditions, EU_SILC).

Optimism was measured using six questions from the revised version of the Life Orientation Test (LOT-R)^76^. Statements such as “In uncertain times, I usually expect the best” were rated on a 5-point scale from “strongly disagree” to “strongly agree”. Cronbach’s α was 0.70.

Self-Efficacy was measured by a short version of the General Self-Efficacy Scale (GSE) developed for use in MoBa^70^. It consists of five statements, e.g. “I can always manage to solve difficult problems if I try hard enough”, scored on a 4-point scale from “not correct” to “highly correct”. Cronbach’s α was 0.86.

### Data analysis

First, we estimated the phenotypic correlation between the two resilience measures. Then, we used biometric analyses to estimate the relative contribution of genetic and environmental factors to individual differences in type 1 and type 2 resilience, and their association, by means of twin modelling.

In classical twin models, the difference between MZ and DZ twins is utilised to break down the phenotypic variation into variance due to additive genetic factors (A) or dominant genetic factors (D), shared environmental factors (C), and individual-specific environmental sources (E)^77^. In our data, the DZ-correlation was not more than half the MZ-correlation, which indicates a genetic influence based on additive genetic factors, supporting the use of an ACE-model. “A” contributes twice as much to the correlations between MZ twins (100% shared genetic material) than DZ twins (50% shared genetic material). “C” contributes equally to the correlations between MZ and DZ co-twins (reared together). Finally, “E” reflects environmental influences not shared by twins and, consequently, indicates differences between co-twins (and measurement errors).

Using the OpenMx package^78–81^ in R^82^, five different nested models were run for the resilience indicators with a full “ACE” model including sex differences (i.e., “common sex limitation”) as starting point (Model 1). Model 2 involved parameters for A and E, and quantitative sex differences, allowing the estimates of genetic and environmental contributions to differ between males and females. Model 3 and 4 involved scalar sex limitations, allowing variance differences across sex, but constraining the standardized effects (i.e., heritability) to be equal^83^. Model 3 involved all three variance components (ACE), while Model 4 excluded the C component. Model 5 and 6 specified no sex limitations, where Model 5 involved all ACE parameters and model 6 omitted the C component. The best fitting model was evaluated by (1) testing the differences in − 2 log likelihood between the models (Δχ^2^df), where fewer parameters are preferable if they do not involve a significant deterioration in fit, and (2) the Akaike’s Information Criteria (AIC), which provides a summary of index of both parsimony and fit (Δχ^2^–2Δdf)^84^. To analyse the bivariate data structure, the Cholesky decomposition approach^85^ was utilised. The correlations between the genetic variations (r_g_) and the environmental variations (r_e_) were constrained to be equal for both sexes.

Finally, we investigated predictors of type 1 and type 2 resilience, using a co-twin control (CTC) design to control for potential genetic and shared environment confounding. In a CTC design, the individual level association between exposure (to predictor) and outcome (e.g., resilience) is compared to the corresponding within-MZ exposure-outcome association^86^. If the association between exposure and outcome remains equally strong when analysed within MZ pairs, the effect is seen as reflecting individual-specific (non-shared) environmental influences. Conversely, if the association disappears in the within pair analyses, the initial association is most likely due to genetic or shared environment confounding^86^.

There are different ways to approach this design. We used linear mixed models, with fixed effects and random intercepts, controlling for the within-pair mean score among the MZ twins (lme4 package in R)^87^. Four different models were tested. First, univariate regression analyses were completed for each variable separately, with control for biological sex and age (Model 1). Then the co-twin control was included in the univariate regression analyses, with controls for the pair level (random intercept) in addition to biological sex and age (Model 2). After this, a model with the additional control for the other resilience measure was run to explore potentially unique predictors for each resilience indicator (Model 3). Lastly, a model based on multivariate linear regression was conducted (Model 4). This model included all the significant variables from Model 2, with control for genetic and shared environment confounding, biological sex and age.

Generally, across all variables we observed an average missingness of 3.5%. Given that some predictors require being in a couple relationship, Relationship Satisfaction and Attachment had missingness of 18% and 10% respectively. For the remaining variables, average missingness was 1.4%. The biometric models (and results in Table 3) are based on the Full Information Maximum Likelihood (FIML) estimator, which uses all available information, thus with N = 1987 twins. The models in table 4 and 5 include only MZ twins. The linear mixed models (Model 2–4) also only use pairs in which both twins have responded. Thus, given varying missingness across predictors, N varies between 415 (type 1 resilience, multivariate Model 4) and 888 (Model 1). The analyses are based on a long-file, where individuals represent cases, nested within pairs, and in which the pair level is controlled for by including random intercepts in the models.

## Supplementary Information

Supplementary Information.

## Data Availability

The data analysed in this study may be requested from the Norwegian Twin Registry. However, restrictions apply to its availability. Information about data access is available on this link: https://www.fi.no/en/studies/norwegian-twin-registry.

## Code availability

All R codes used are available from the first author upon request.
