# Supplementary material for: Introducing two types of psychological resilience with partly unique genetic and environmental sources
Source: Sci Rep. 2021 Apr 21;11:8624. doi: 10.1038/s41598-021-87581-5 (PMC8060303; doi:10.1038/s41598-021-87581-5)
Supplement: Supplementary file 1 — Supplementary Information. [file 41598_2021_87581_MOESM1_ESM.pdf]

Supplementary Information to

Introducing Two Types of Psychological Resilience with Partly Unique Genetic and Environmental Sources

Live Skow Hofgaard* [1]
Ragnhild Bang Nes [1, 2]
Espen Røysamb [1, 2]

[1] Promenta Research Centre, Department of Psychology, University of Oslo, Norway.
[2] The Norwegian Institute of Public Health, Norway.
* Corresponding author: Live Skow Hofgaard, mail: l.s.hofgaard@psykologi.uio.no

Table of Contents

Supplementary Tables 1
    Table S1 2
Supplementary Figures 3
    Figure S1 3
    Figure S2 3
    Figure S3 4
    Figure S4 4

Supplementary Tables

Table S1

Pearson correlations between all variables

Variable	1	2	3	4	5	6	7	8	9	10	11	12	13	14	15	16	17	18
1. Educational Background																		
2. Self-Rated Health	.214**																	
3. Physical Activity	.126**	.221**																
4. Meaning in Life	.198**	.362**	.068**															
5. Positive Affect	.093**	.279**	.086**	.552**														
6. Loneliness	-.041	-.173**	-.033	-.427**	-.379**													
7. Relationship satisfaction	-.005	.152**	.031	.388**	.414**	-.396**												
8. Social Support	.093**	.067**	.097**	.180**	.218**	-.276**	.098**											
9. Optimism	.224**	.284**	.070**	.414**	.453**	-.343**	.286**	.207**										
10. Trust	.162**	.148**	.089**	.274**	.249**	-.273**	.168**	.210**	.344**									
11. Self-efficacy	.160**	.201**	.028	.360**	.370**	-.263**	.211**	.092**	.414**	.164**								
12. Anxious Attachment	-.077**	-.195**	-.057*	-.291**	-.280**	.389**	-.334**	-.117**	-.365**	-.223**	-.230**							
13. Avoidant Attachment	-.061**	-.161**	-.054*	-.360**	-.385**	.430**	-.612**	-.173**	-.350**	-.201**	-.195**	.476**						
14. Type 1 Resilience	.165**	.318**	.066**	.425**	.356**	-.357**	.282**	.093**	.368**	.182**	.311**	-.327**	-.289**					
15. Type 2 Resilience	.180**	.384**	.078**	.610**	.575**	-.352**	.459**	.134**	.383**	.213**	.367**	-.281**	-.361**	.445**				
16. Lifetime Stressors	.054*	-.195**	-.020	-.189**	-.139**	.257**	-.242**	.031	-.104**	-.153**	-.039	.178**	.175**	.000	.000			
17. Satisfaction With Life Scale	.150**	.431**	.073**	.658**	.572**	-.415**	.503**	.106**	.411**	.242**	.356**	-.310**	-.394**	.461**	.912**	-.310**		
18. Symptom Checklist (SCL-8)	-.147**	-.361**	-.056*	-.470**	-.359**	.399**	-.336**	-.071**	-.372**	-.211**	-.310**	.353**	.322**	-.865**	-.393**	.334**	-.534**	
19. Birth Year (age)	.118**	.036	-.022	.030	.056*	-.002	.012	.057*	.094**	.029	.067**	-.042	-.092**	-.043	.017	.101**	-.016	.061**

**. Correlation is significant at the 0.01 level (2-tailed).

*. Correlation is significant at the 0.05 level (2-tailed).

Supplementary Figures

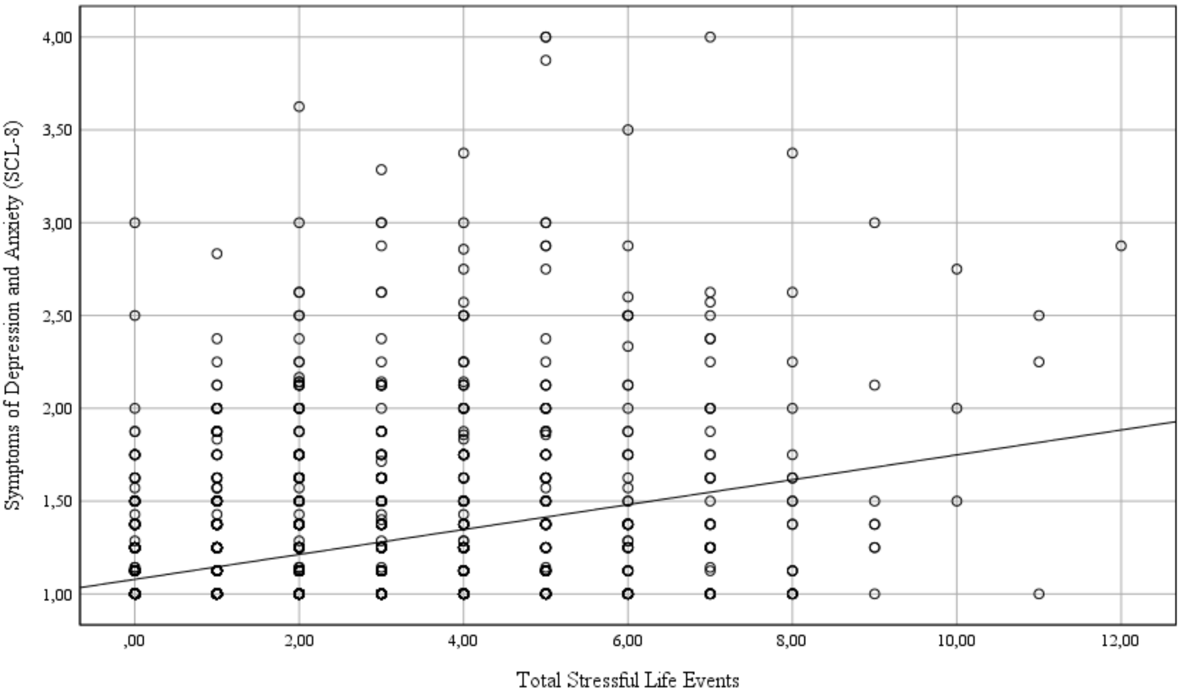

Figure S1: The relationship between symptoms of depression and anxiety (SCL) and total stressful life events experienced. R^2 Linear = .112.

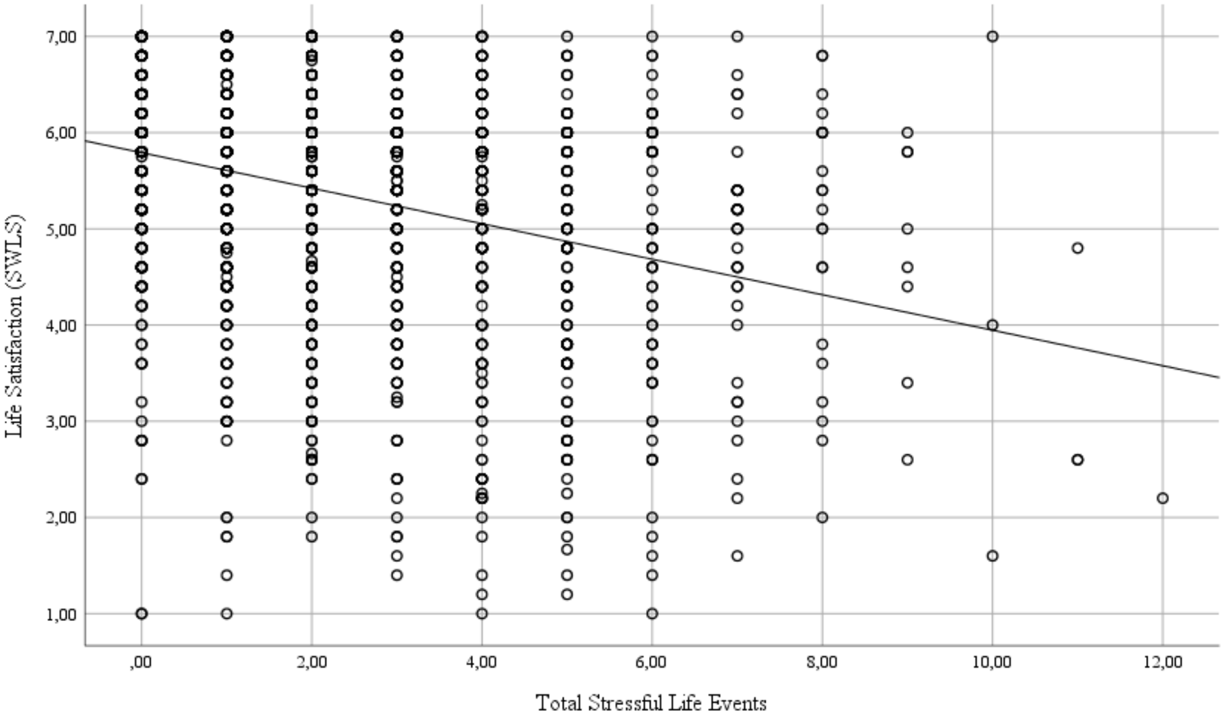

Figure S2: The relationship between life satisfaction (SWLS) and total stressful life events experienced. R^2 Linear = .096.

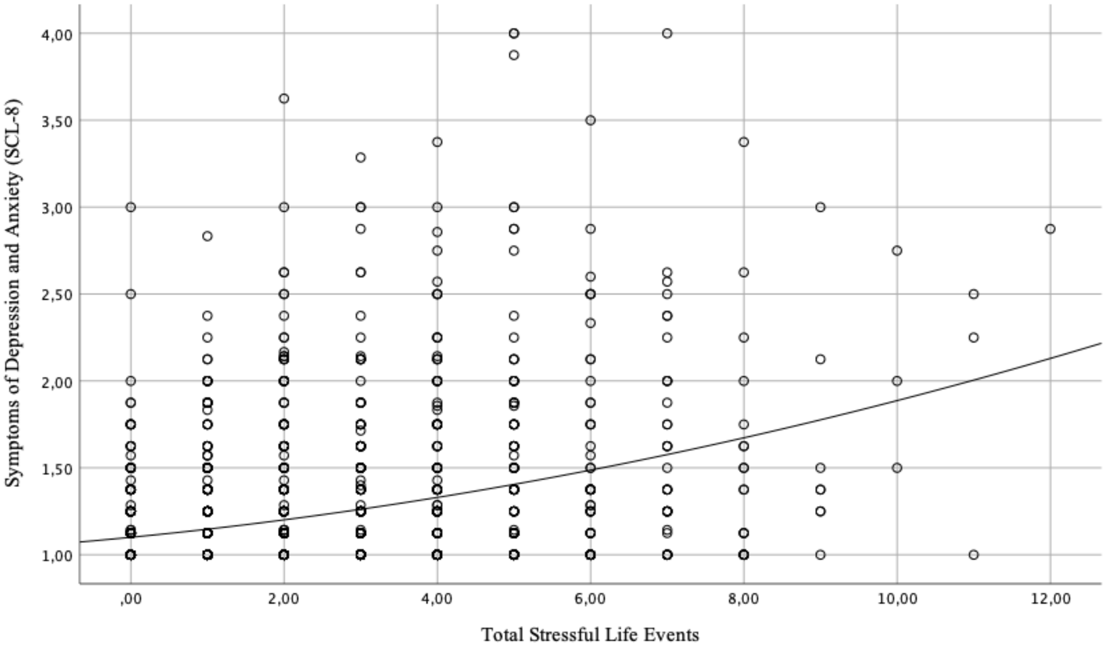

Figure S3: The relationship between symptoms of depression and anxiety (SCL) and total stressful life events experienced. R^2 Quadratic = .114.

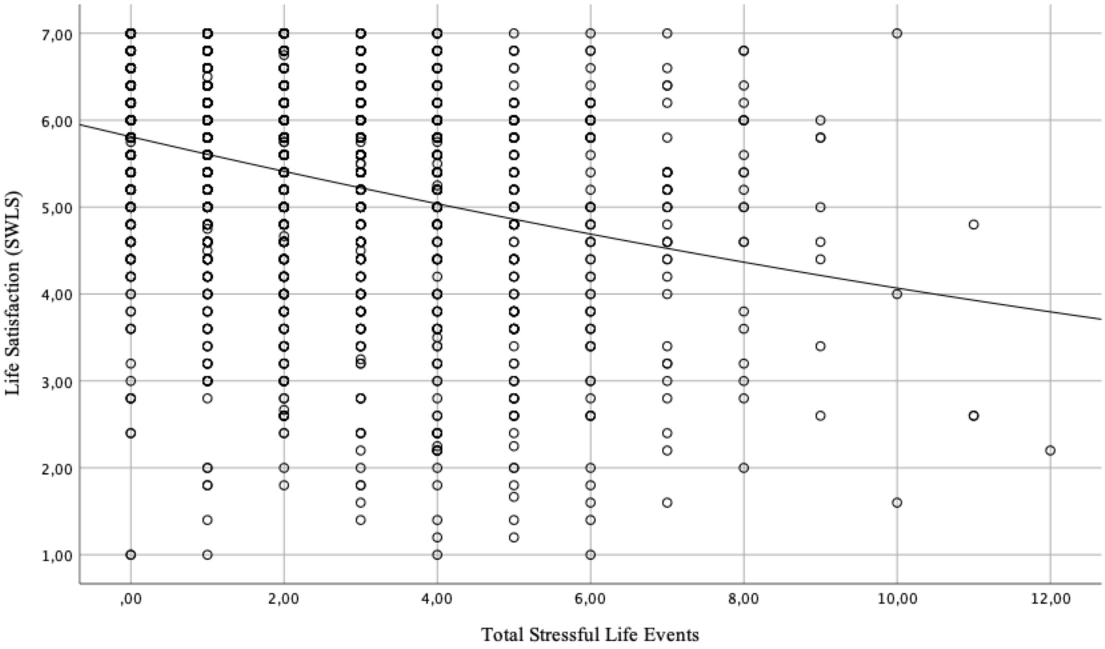

Figure S4: The relationship between life satisfaction (SWLS) and total stressful life events experienced. R^2 Quadratic = .096.
